# Intussusception

**Published:** 1903-02-21

**Authors:** 


### INTUSSUSCEPTION.

During the nine days which followed last Christmas Day Mr. Hugh W. Rigby,[1] who was the surgeon on duty at the London Hospital for that period, had the luck to have seven cases of intussusception admitted under his care. Six of these cases were submitted to operation and of these five recovered, which is distinctly encouraging and should, as he says, serve as a strong argument in favour of early resort to surgical intervention in these too often fatal forms of intestinal obstruction. One case was evidently quite beyond surgical treatment, and died soon after admission into the hospital. Of the six cases operated upon only one was irreducible and gangrenous, the others were reducible, some with ease and some with more or less difficulty. In no case was inflation or injection of fluid by the rectum attempted, the futility of thus wasting valuable time having been amply demonstrated, whilst the cases completely relieved by these methods are few and problematical. In each case an attempt was made to reduce the gut by simply introducing two fingers of one hand into the abdomen and manipulating the gut with the other hand upon the abdominal wall. Complete reduction by this method was, however, found to be impossible, and in every case the cæcum, colon and ileo-cæcal junction had to be brought up to the wound and manipulated outside the abdominal cavity. The difficulty of complete reduction by the first method was found to be due to the mobility of the cæcum, a distinct mesocæcum being present in nearly every case. Great care was taken to prevent prolapse of gut or chilling of that which was necessarily exposed taking place. Mr. Rigby feels sure that the keynote of success in operating on these cases is rapidity. The time occupied from the actual incision of the abdominal wall to the tying of the last suture was noted and did not exceed 15 minutes in any case—one taking 12 minutes and another only 10. Feeding was commenced almost as soon as they had recovered from the anæsthetic effects. This in the absence of vomiting Mr. Rigby regards as an essential point, for in many cases children have been constantly sick for two or perhaps three days before admission, and are so exhausted that to withhold nourishment for a further six or eight hours after operation may turn the balance in the wrong direction. Opium (two minim doses of nepenthe) was given to each child if any restlessness was present.

[1] Lancet, Feb. 7.

